# SARS-CoV-2 Spike Protein Elicits Cell Signaling in Human Host Cells: Implications for Possible Consequences of COVID-19 Vaccines

**DOI:** 10.3390/vaccines9010036

**Published:** 2021-01-11

**Authors:** Yuichiro J. Suzuki, Sergiy G. Gychka

**Affiliations:** 1 Department of Pharmacology and Physiology, Georgetown University Medical Center, Washington, DC 20007, USA; 2 Department of Pathological Anatomy N2, Bogomolets National Medical University, 01601 Kiev, Ukraine; gychka59@gmail.com

**Keywords:** cell signaling, coronavirus, COVID-19, SARS-CoV-2, spike protein, vaccine

## Abstract

The world is suffering from the coronavirus disease 2019 (COVID-19) pandemic caused by severe acute respiratory syndrome coronavirus 2 (SARS-CoV-2). SARS-CoV-2 uses its spike protein to enter the host cells. Vaccines that introduce the spike protein into our body to elicit virus-neutralizing antibodies are currently being developed. In this article, we note that human host cells sensitively respond to the spike protein to elicit cell signaling. Thus, it is important to be aware that the spike protein produced by the new COVID-19 vaccines may also affect the host cells. We should monitor the long-term consequences of these vaccines carefully, especially when they are administered to otherwise healthy individuals. Further investigations on the effects of the SARS-CoV-2 spike protein on human cells and appropriate experimental animal models are warranted.

## 1. Introduction

The world is suffering from the coronavirus disease 2019 (COVID-19) pandemic caused by severe acute respiratory syndrome coronavirus 2 (SARS-CoV-2), a positive-sense, single-stranded RNA virus [1,2]. As of the end of December 2020, over 80 million people have been infected with SARS-CoV-2, causing 1.8 million deaths worldwide. SARS-CoV-2 uses its viral membrane fusion protein, known as a spike protein, to bind to angiotensin converting enzyme 2 (ACE2) as a ‘receptor’ in order to enter human host cells [3,4], causing severe pneumonia and acute respiratory distress syndrome (ARDS) [5]. Elderly patients with cardiovascular disease are particularly susceptible to developing serious COVID-19 conditions that in some cases lead to death, while young and healthy individuals are largely resistant to developing severe symptoms [1,6,7]. As COVID-19 continues to cause serious health, economic, and sociological problems, the world awaits the widespread rollout of effective vaccines that may end this pandemic.

**Figure 1:**
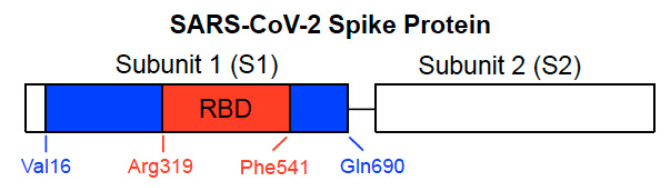
Structure of SARS-CoV-2 spike protein. The spike protein consists of Subunit 1 (S1) and Subunit 2 (S2). The S1 subunit contains the receptor-binding domain (RBD) that binds to ACE2 of the host cell membrane. The S2 subunit is responsible for fusion. In our previous study described in Section 3 and Section 5, we used full-length S1 (Val16-Gln690) depicted with blue and red regions and the RBD only-containing protein (Arg319-Phe541) shown in red of the SARS-CoV-2 spike protein (GenBank Accession Number: QHD43416.1).

The SARS-CoV-2 spike protein, a class I viral fusion protein, is critical to initiating the interactions between the virus and the host cell surface receptor, facilitating viral entry into the host cell by assisting in the fusion of the viral and host cell membranes. This protein consists of two subunits: Subunit 1 (S1) that contains the ACE2 receptor-binding domain (RBD) and Subunit 2 (S2) that plays a role in the fusion process [3,4] (Figure 1). The SARS-CoV-2 spike protein is the major target for the development of COVID-19 vaccines.

## 2. Development of Spike Protein-Based COVID-19 Vaccines

The remarkably rapid development of vaccines and therapeutics for COVID-19 in 2020 has been due to effective collaborations between governments and the private sector. On 9 November 2020, Pfizer and BioNTech announced that their mRNA-based vaccine candidate, BNT162b2, is more than 90% effective against COVID-19 [8]. This was welcome news in that it revealed that effective vaccines may soon become available. BNT162b2 encodes the SARS-CoV-2 spike protein to elicit virus-neutralizing antibodies [9,10]. More specifically, it encodes the full-length spike protein of SARS-CoV-2 with two amino acids mutated to proline in the S2 subunit to maintain the prefusion conformation, while its sister vaccine BNT162b1 (also from Pfizer/BioNTech) encodes only the RBD of the SARS-CoV-2 spike protein, trimerized by the addition of a T4 fibritin foldon domain [9,10,11]. Clinical trials have demonstrated that neither BNT162b1 [11] nor BNT162b12 [9,10] exhibit serious short-term adverse effects. On 10 December 2020, the results of a large clinical trial for BNT162b were published, showing that this vaccine conferred 95% protection in persons 16 years of age or older [12]. Long-term consequences of these vaccines are, however, unknown.

Another promising vaccine, mRNA-1273 by Moderna, is also an RNA vaccine that encodes the full-length SARS-CoV-2 spike protein [13]. Viral vector-based vaccines such as AZD1222 by AstraZeneca, which uses a non-replicating chimpanzee adenovirus vector [14], Ad26.COV2.S by Johnson & Johnson, a non-replicating adenovirus 26-based system [15], and Gam-COVID-Vac (Sputnik V) by Gamaleya Research Institute of Epidemiology and Microbiology [16], all express the SARS-CoV-2 spike protein. NVX-CoV2373 (Novavax), a recombinant protein-based vaccine [17], is also the full-length SARS-CoV-2 spike protein. These vaccines as well as many others under development [18,19,20] introduce the SARS-CoV-2 spike protein into our body, so that the production of antibodies and immunity against SARS-CoV-2 are stimulated.

## 3. SARS-CoV-2 Spike Protein Elicits Cell Signaling in Human Cells

It was found that the treatment of cultured primary human pulmonary artery smooth muscle cells (SMCs) or human pulmonary artery endothelial cells with the recombinant SARS-CoV-2 spike protein S1 subunit is sufficient to promote cell signaling without the rest of the viral components [21]. Furthermore, our analysis of the postmortem lung tissues of patients who died of COVID-19 has determined that these patients exhibited pulmonary vascular wall thickening, a hallmark of pulmonary arterial hypertension (PAH) [21]. Based on these results, we proposed that the SARS-CoV-2 spike protein (without the rest of the viral components) triggers cell signaling events that may promote pulmonary vascular remodeling and PAH as well as possibly other cardiovascular complications [21,22].

In our cell culture experiments, two recombinant SARS-CoV-2 spike proteins, both of which contain the RBD, were studied [21]. The full-length S1 subunit protein contains most of the S1 subunit (Val16–Gln690), while the RBD S1 subunit protein only contains the RBD region (Arg319–Phe541), as shown in Figure 1. Cultured primary human pulmonary artery SMCs and human pulmonary artery endothelial cells were treated with these proteins for 10 min. We found, using the phospho-specific MEK antibody, that the recombinant full-length S1 subunit of SARS-CoV-2 alone at a concentration as low as 130 pM activated MEK, the activator of extracellular signal-regulated kinase (ERK) and a well-known signal transduction mechanism for cell growth [23]. By contrast, such activation of cell signaling by the spike protein did not occur in rat pulmonary artery SMCs [21].

**Figure 2:**
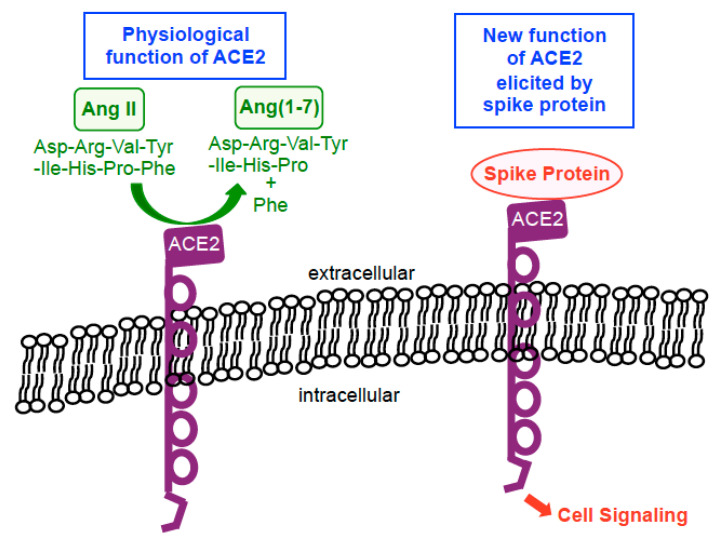
Biological functions of ACE2. In physiological situations, ACE2 functions as a carboxypeptidase enzyme that catalyzes the hydrolysis of angiotensin II (Ang II) into Ang(1–7) by cleaving off a phenylalanine (Phe). In the presence of the spike protein, this enzyme becomes a membrane receptor for cell signaling that uses the spike protein as a ligand for its activation.

While ACE2 is now well known as a ‘receptor’ to which the SARS-CoV-2 spike protein binds on human host cells in order to facilitate the membrane fusion and gain viral entry, the usual physiological function of ACE2 is not to serve as a membrane receptor to transduce intracellular signals. ACE2 is a type I integral membrane protein that functions as a carboxypeptidase, cleaving angiotensin II to angiotensin (1–7) and regulating blood pressure [24,25] (Figure 2). However, ten years ago, Chen et al. [26] reported the intriguing findings showing that ACE2 acts as a membrane receptor for cell signal transduction in response to the spike protein of SARS-CoV (now also known as SARS-CoV-1, the virus that caused the SARS outbreak in 2002–2004) in the human lung alveolar epithelial cell line, A549. The spike protein of SARS-CoV-1 is 76–78% identical to that of SARS-CoV-2 [27]. In their study, it was shown that the binding of the full-length spike protein to ACE2 triggered the casein kinase II-dependent activation of activator protein-1 (AP-1) transcription factor and subsequent gene transcriptional events [26]. Their finding on SARS-CoV-1 [26] and ours on SARS-CoV-2 [21] indicate that the spike protein remarkably functionally converts ACE2 (that is normally a peptidase enzyme) into a membrane receptor for cell signaling that uses the spike protein as a ligand for its activation (Figure 2).

Kuba et al. [28] showed that the injection of mice with recombinant SARS-CoV-1 spike protein reduced the ACE2 expression and worsened the acid-induced lung injury. In mice with an acid-induced lung injury, the recombinant SARS-CoV-1 spike protein dramatically increased angiotensin II, and the angiotensin receptor inhibitor losartan attenuated the spike protein-induced enhancement of lung injury [28]. Thus, these in vivo studies demonstrated that the spike protein of SARS-CoV-1 (without the rest of the virus) reduces the ACE2 expression, increases the level of angiotensin II, and exacerbates the lung injury.

The SARS-CoV-2 spike protein without the rest of the viral components has also been shown to activate cell signaling by Patra et al. [29]. The authors reported that the full-length SARS-CoV-2 spike protein expressed by the means of transient transfection, either in the human lung alveolar epithelial cell line A549 or in the human liver epithelial cell line Huh7.5, activated NF-κB and AP-1 transcription factors as well as p38 and ERK mitogen-activated protein kinases, releasing interleukin-6. This cell signaling cascade was found to be triggered by the SARS-CoV-2 spike protein downregulating the ACE2 protein expression, subsequently activating the angiotensin II type 1 receptor [29]. These experiments using transient transfection may reflect the intracellular effects of the spike protein that could be triggered by the RNA- and viral vector-based vaccines.

These results collectively reinforce the idea that human cells are sensitively affected by the extracellular and/or intracellular spike proteins though the activation of cell signal transduction.

## 4. Pulmonary Hypertension

PAH is a serious disease without a cure that can affect males and females of any age including children. The increased pulmonary vascular resistance in PAH results in right heart failure and subsequently death. Patients diagnosed with PAH only live for 2–3 years from the time of diagnosis on average if untreated [30,31]. Even with currently available therapies, only 60–70% of PAH patients survive for three years [32,33,34,35]. PAH is hard to detect because its symptoms (e.g., shortness of breath, fatigue, and dizziness) are similar to those of other common non-life threatening conditions, and the official diagnosis for PAH must be made through invasive right heart catheterization [36]. Endothelial dysfunction is a common feature of patients with PAH and COVID-19 [37,38].

PAH “outbreaks” have occurred in association with exposure to certain drugs or toxins [39]. A major outbreak of PAH occurred in 1965 and was associated with aminorex, a weight-loss stimulating drug [39,40]. Approximately 0.2% of people who took this drug developed PAH [40]. An epidemic was observed two years after the introduction of aminorex, and half of the patients died 10 years after the epidemic [39].

We studied pulmonary vessels of COVID-19 patients and those of H1N1 influenza-infected patients who died of ARDS [21]. The pulmonary arteries of postmortem COVID-19 patient lungs consistently exhibited histological characteristics of vascular wall thickening, mainly due to the hypertrophy of the tunica media. Detailed pathological analysis revealed that the boundaries between the vessels and the surrounding lung parenchyma have lost clarity, the SMCs of the middle lining of the arteries have enlarged, the nuclei of SMCs have swollen, and vacuoles have been generated in the cytoplasm of SMCs [21]. A morphometric analysis determined that the median pulmonary vascular wall thickness values were 15.4 μm for the COVID-19 patients and 6.7 μm for the influenza patients, and these values were significantly different from each other [21]. Pulmonary vascular wall thickening in COVID-19 patients was also observed on the computed tomography scan of the chest [41,42]. Thus, these results together indicated that COVID-19 is associated with pulmonary vascular wall thickening. Investigations on whether this pulmonary vascular wall thickening is related to clinically significant PAH and the role of the spike protein in the pathogenesis of PAH are warranted.

## 5. RBD Only-Containing SARS-CoV-2 Spike Protein Does Not Elicit Cell Signaling in Human Cells

In contrast to the full-length spike protein [26,29] or the full-length SARS-CoV-2 spike protein S1 subunit [21], we found that the RBD only-containing protein (Figure 1) did not promote cell signaling. Our Western blotting results monitoring the MEK activation showed that the mean ± SEM phosphorylated MEK to MEK protein ratio values were 0.05 ± 0.003 (untreated), 1.9 ± 0.07 (treated with the full-length S1 protein), and 0.05 ± 0.003 (treated with the RBD only-containing protein) for human pulmonary artery SMCs; and 0.09 ± 0.006 (untreated), 0.90 ± 0.06 (treated with the full-length S1 protein), and 0.10 ± 0.003 (treated with the RBD only-containing protein) for human pulmonary artery endothelial cells [21].

The different effects of the full-length S1 and RBD only-containing proteins may be important considering that BNT162b2 and many other COVID-19 vaccines express the full-length spike protein, while the BNT162b1 vaccine encodes only the RBD region [9,10,11,12,13,14,15,16,17,18,19,20]. There are some other RBD-based COVID-19 vaccines being developed as well [43]. It is possible that the RBD-based vaccines are less immunogenic, but may not affect the host cells. Thus, they may be less risky considering potential long-term adverse effects. However, in the in vivo study of the SARS-CoV-1 spike protein described above [28], a deletion mutant that only contained the RBD also worsened the acid-induced lung failure, like the full-length spike protein. Thus, further work is needed to understand effects of the full-length spike protein and the RBD-only containing protein in various biological processes.

## 6. Discussion

It is generally thought that the sole function of viral membrane fusion proteins is to allow the viruses to bind to the host cells for the purpose of viral entry into the cells, so that the genetic materials can be released and the viral replication and amplification can take place. However, recent observations suggest that the SARS-CoV-2 spike protein can by itself trigger cell signaling that can lead to various biological processes. It is reasonable to assume that such events, in some cases, result in the pathogenesis of certain diseases.

**Figure 3:**
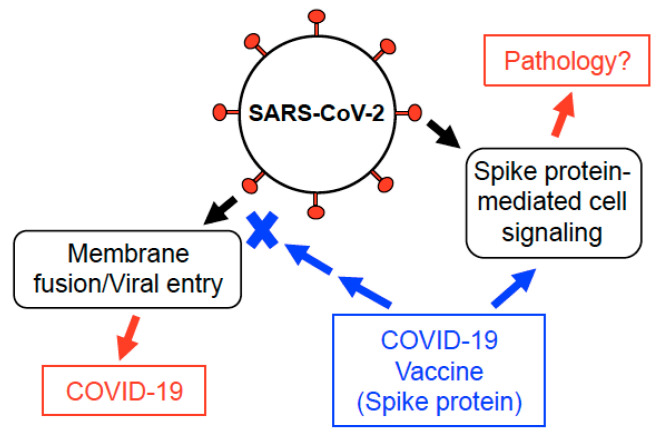
Possible actions of the SARS-CoV-2 spike protein. The SARS-CoV-2 spike protein of the intact virus targets ACE2 of the host cells to facilitate the membrane fusion and the viral entry. The SARS-CoV-2 spike protein also elicits cell signaling in human cells [21,29]. COVID-19 vaccines introduce the spike protein into the human body. In addition to eliciting an immune response that suppresses the viral entry, the spike protein produced by the COVID-19 vaccines may also affect the host cells, possibly triggering adverse events. Further investigations addressing this possibility are warranted.

Our laboratory only tested the effects of the SARS-CoV-2 spike protein in lung vascular cells and those implicated in the development of PAH. However, this protein may also affect the cells of systemic and coronary vasculatures, eliciting other cardiovascular diseases such as coronary artery disease, systemic hypertension, and stroke. In addition to cardiovascular cells, other cells that express ACE2 have the potential to be affected by the SARS-CoV-2 spike protein, which may cause adverse pathological events. Thus, it is important to consider the possibility that the SARS-CoV-2 spike protein produced by the new COVID-19 vaccines triggers cell signaling events that promote PAH, other cardiovascular complications, and/or complications in other tissues/organs in certain individuals (Figure 3). We will need to monitor carefully the long-term consequences of COVID-19 vaccines that introduce the spike protein into the human body. Furthermore, while human data on the possible long-term consequences of spike protein-based COVID-19 vaccines will not be available soon, it is imperative that appropriate experimental animal models are employed as soon as possible to ensure that the SARS-CoV-2 spike protein does not elicit any signs of the pathogenesis of PAH or any other chronic pathological conditions.

## 7. Conclusions

In conclusion, the recent advancement in the SARS-CoV-2 spike protein-based COVID-19 vaccine development is exciting and has shed light on how to end the current pandemic. These vaccines should benefit elderly people with underlying conditions if they do not exhibit any acute adverse events. However, we need to consider their long-term consequences carefully, especially when they are administered to otherwise healthy individuals as well as young adults and children. In addition to evaluating data that will become available from SARS-CoV-2 infected individuals as well as those who received the spike protein-based vaccines, further investigations of the effects of the SARS-CoV-2 spike protein in human cells and appropriate animal models are warranted.
